# Genome-based reclassification of Parabacteroides chartae as a later heterotypic synonym of Macellibacteroides fermentans

**DOI:** 10.1099/ijsem.0.006927

**Published:** 2025-09-25

**Authors:** Samuel L. Miller, Noha H. Youssef, Mostafa S. Elshahed

**Affiliations:** 1 Department of Microbiology and Molecular Genetics, Oklahoma State University, Stillwater, OK, USA

**Keywords:** *Bacteroidales*, *Macellibacteroides fermentans*, *Parabacteroides chartae*, *Porphyromonadaceae*, *Tannerellaceae*

## Abstract

Strain PK034 was isolated from a municipal wastewater treatment plant in Stillwater, OK, USA. A comparison of 16S rRNA gene sequences revealed ≥99.4% sequence similarity to the type strains of *Macellibacteroides fermentans* (isolated from an up-flow anaerobic filter) and *Parabacteroides chartae* (isolated from wastewater from a paper mill), suggesting their close relatedness and the possibility of these strains belonging to the same species. The genus *Macellibacteroides* currently contains a single species, while the genus *Parabacteroides* currently contains 13 species. Phylogenetic analysis using the 16S rRNA gene, whole-genome phylogenomic analysis and overall genomic-based relatedness indices indicated that PK034 and the type strains of *M. fermentans* and *P. chartae* shared average nucleotide identity and digital DNA–DNA hybridization values >95% and >70%, which are the currently accepted thresholds for species-level delineation. Furthermore, such analysis also placed PK034 and the type strains of *M. fermentans* and *P. chartae* as a distinct branch within the *Tannerellaceae* family of the *Bacteroidales* order, clustering separately from all other species of the genus *Parabacteroides*. Comparison of phenotypic and physiological traits revealed a high level of shared characteristics and physiological optima between all isolates. Thus, it is proposed that *P. chartae* NS31-3^T^ has been erroneously classified as a member of the genus *Parabacteroides*, that strains PK034, *M. fermentans* and *P. chartae* constitute the same species and that *P. chartae* Tan *et al.* 2012 should be taken as a later heterotypic synonym of *M. fermentans* Jabari *et al.* 2012.

## Introduction

The phylum *Bacteroidota* currently encompasses six classes and seven orders (https://lpsn.dsmz.de/phylum/Bacteroidota, April 2025). Members of the *Bacteroidota* are Gram-stain-negative rods that do not form endospores and are predominantly obligate anaerobes [1]. Taxa in the phylum *Bacteroidota* are widely distributed in a wide range of habitats, including marine [2,3], freshwater [4,5] and terrestrial habitats [6,7], and are highly abundant in the human alimentary canal [8,9]. *Bacteroidota* is a keystone phylum concerning the composition of the human gut microbiome [10] as well as the microbiome of several non-human mammals, such as cows [11], goats [12] and whales [13]. Additionally, these taxa are highly abundant in industrial environmental sources, such as wastewater treatment plants [14,15].

A targeted isolation approach was undertaken to recover esculin-positive, non-spore-forming obligate anaerobes from wastewater treatment plants. Strain PK034 was isolated from an anoxic enrichment of return-activated sludge from the Stillwater, OK, USA, wastewater treatment plant (36.0986° N 97.0147° W) on 27 September 2024. Briefly, 1.0 ml of return-activated sludge was added to modified medium 2 with pectin as a sole carbon substrate, as previously described [16], and incubated at 37 °C for 2 weeks in an anaerobic chamber (Coy Laboratory Products, Ann Arbor, MI, USA); following incubation, an aliquot from the enrichment was plated onto *Bacteroides* Bile Esculin (BBE) agar (Anaerobe Systems, Morgan Hill, CA, USA) to select for esculin-positive bacterial colonies.

## Phylogenetic assessment

A near full-length 16S rRNA gene sequence for strain PK034 was obtained using Sanger sequencing [17] using the universal primers 27F (5′-AGAGTTTGATCCTGGCTCAG-3′) and 1492R (5′-TACGGYTACCTTGTTACGACTT-3′). Initial assessment against described taxa (using EzBioCloud platform; https://www.ezbiocloud.net/ [18]) revealed high sequence similarity values (99.5 and 99.4%) with 16S rRNA gene sequences from two validly published taxa: *Macellibacteroides fermentans* (HQ020488) and *Parabacteroides chartae* (JN029805). *M. fermentans* currently constitutes the only species within the genus *Macellibacteroides* (https://lpsn.dsmz.de/genus/Macellibacteroides, April 2025). On the other hand, the genus *Parabacteroides* currently includes 13 species, including *P. chartae* (https://lpsn.dsmz.de/genus/*Parabacteroides*, April 2025).

More detailed 16S rRNA gene phylogenetic analysis was conducted by downloading closely related species identified with the EzBioCloud server [18]. Sequences were aligned using muscle [19], and a phylogenetic tree was reconstructed using the maximum-likelihood method [20] and the Tamura–Nei substitution model [21] with 1,000 bootstrap replications. The tree was visualized in MEGA11 [22]. As well, since the Hsp60 locus has been previously suggested as a useful phylogenetic marker for Gram-negative anaerobic rods and successfully applied for members of the *Bacteroidota* [23,24], we conducted a phylogenetic analysis using Hsp60 proteins of representatives of *Tannerellaceae* (*Parabacteroides*, *Tannerella* and *Macellibacteroides*) and *Porphyromonadaceae* (*Porphyromonas* and *Porphyromonas*_A). Hsp60 protein sequences were downloaded from GenBank, aligned using MAFFT [25], and the alignment was used to construct a maximum-likelihood phylogenetic tree in FastTree [26].

**Fig. 1.**
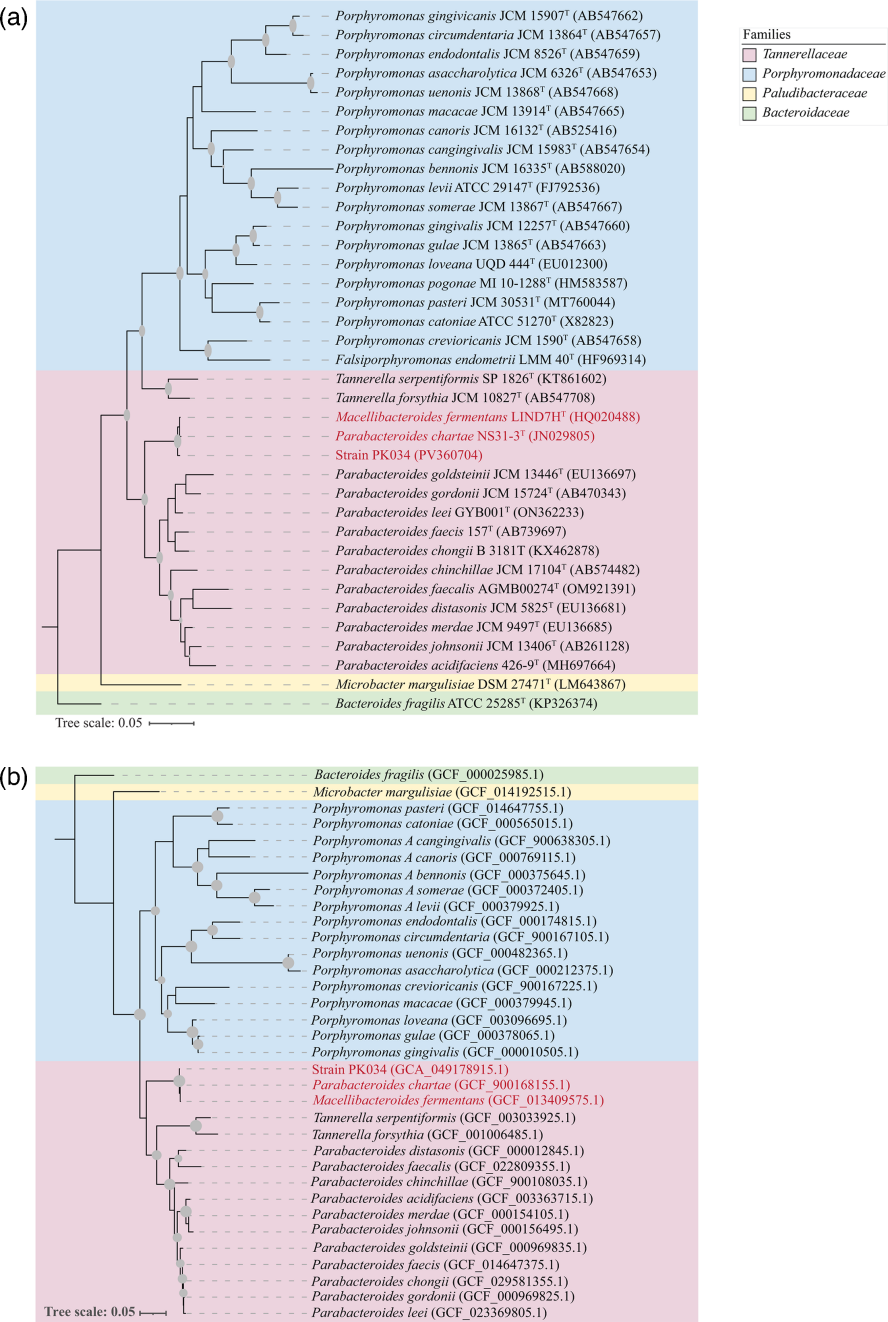
Phylogenetic tree based on 16S rRNA gene (**a**) and Hsp60 (**b**) sequences showing the relationship among PK034, *M. fermentans* and *P. chartae* among other species of the genus *Parabacteroides*. Bar, 0.05 substitutions per site.

Phylogenetic analysis placed strain PK034 as a member of the *Bacteroidota*, sharing high 16S rRNA gene sequence similarity (≥99.4%) with its closest relatives, *M. fermentans* and *P. chartae* (Fig. 1a, Table 1). Such values exceed the recommended species-level thresholds of 98.6%. Similar results were also observed using Hsp60 as the phylogenetic marker (Fig. 1b). Pairwise Hsp60 similarity ranged between 99.63% (for *P. chartae* and strain PK034) and 99.82% (for both *M. fermentans* and strain PK034, and *M. fermentans* and *P. chartae*).

**Table 1.** rRNA gene sequence similarity (%) for PK034, *M. fermentans*, *P. chartae* and other *Parabacteroides* spp

16S rRNA gene similarity (%)				
Taxa	**16S rRNA gene accession**	**PK034**	***M. fermentans***	***P. chartae***
**PK034**	PV360704	100.0	99.5	99.4
***M. fermentans***	HQ020488	99.5	100.0	99.8
***P. chartae***	JN029805	99.4	99.8	100.0
*P. acidifaciens*	MH697664	93.1	93.1	93.1
*P. chinchillae*	AB574482	91.3	91.3	91.2
*P. chongii*	KX462878	93.0	92.9	92.8
*P. distasonis*	EU136681	90.2	90.0	90.0
*P. faecalis*	OM921391	88.4	88.4	88.5
*P. faecis*	AB739697	93.5	93.3	93.4
*P. goldsteinii*	EU136697	93.0	92.9	93.0
*P. gordonii*	AB470343	92.3	92.2	92.3
*P. johnsonii*	AB261128	91.8	92.1	92.1
*P. leei*	ON362233	93.5	93.6	93.5
*P. merdae*	EU136685	92.5	92.6	92.4

## Phylogenomic and genome-based assessment

To further clarify the taxonomic relationship between PK034 and the type strains of *M. fermentans* and *P. chartae*, the genome of PK034 was subjected to whole-genome sequencing. Cell biomass was harvested from an overnight culture and stored in DNA/RNA Shield (Zymo, Irvine, CA, USA). DNA extraction, sequencing and annotation were conducted using the services of a commercial provider (Plasmidsaurus, Eugene, OR, USA). genomic DNA (gDNA) was extracted with the Quick-DNA Fungal/Bacterial Miniprep Kit (Zymo, Irvine, CA, USA). Oxford Nanopore Technologies long-read libraries were prepared using amplification-free methods with v14 chemistry, then sequenced using an R10.4.1 flow cell. Default parameters were used for all software unless otherwise specified. Reads were filtered with Filtlong v0.2.1 (https://github.com/rrwick/Filtlong), using a minimum Phred score of 15 and a minimum length of 1,000 bps. The genome was assembled and polished using Flye v2.9.1 [27] and Medaka v1.8.0 (https://github.com/nanoporetech/medaka), respectively. Assembly completeness and contamination were assessed using CheckM v1.2.2 [28]. Genome annotation was performed using the National Center for Biotechnology Information Prokaryotic Genome Annotation Pipeline v6.7 [29].

To delineate species boundaries between all three isolates, overall genomic relatedness indices (OGRIs) between PK034 (PRJNA1240326) and the type strains of *M. fermentans* (GCF_013409575.1) and *P. chartae* (GCF_900168155.1) were assessed according to the most recent proposed minimal standards for the use of genome data for the taxonomy of prokaryotes [30]. For such purpose, average nucleotide identity (ANI) [18,31] and digital DNA–DNA hybridization values (dDDH) [32,33] were computed. Genomic data were also used to determine the relationship between these three isolates and all other members of the genus *Parabacteroides*. For such purpose, the genus-specific delineation indices average amino acid identity (AAI) and percentage of conserved proteins (POCP) were calculated using the AAI calculator [34] and a custom shell script based on previously defined parameters [35]. No similar analysis was feasible for *M. fermentans*, since it represents the only member of the genus *Macellibacteroides*.

Whole-genome-based phylogenomic analysis was performed using the Genome Taxonomy Database (GTDB) Toolkit [36] using a concatenated alignment of 120 single-copy genes [37]. The whole-genome phylogenomic tree was constructed using the maximum-likelihood approach [20] in RAxML using the PROTGAMMABLOSUM62 model and default parameters [38] and was visualized using the Interactive Tree of Life v6 [39].

The genome of PK034 was sequenced to 101× depth. The genome was 4.3 Mb in size, with a G+C content ratio of 41.5 mol%, arranged in three contigs with an N50 of 2.1 Mb, a high level of completion of 98.7% and a low level of contamination of 1.5%. The genome size and G+C content ratio between strain PK034 and the type strains of *M. fermentans* and *P. chartae* were highly similar (Table 2).

**Table 2.** Comparison of genomic features for PK034, *M. fermentans* and *P. chartae*

Characteristic	PK034	*M. fermentans*	*P. chartae*
**Genome accession**	PRJNA1240326	GCF_013409575.1	GCF_900168155.1
**Genome size (Mb**)	4.3	3.9	3.9
**Number of contigs**	3	10	44
**G+C content (mol%**)	41.5	42	41.5
**Protein-coding genes**	3,406	3,155	3,137
**tRNA genes**	72	70	60
**rRNA genes**	20	18	10

**Fig. 2.**
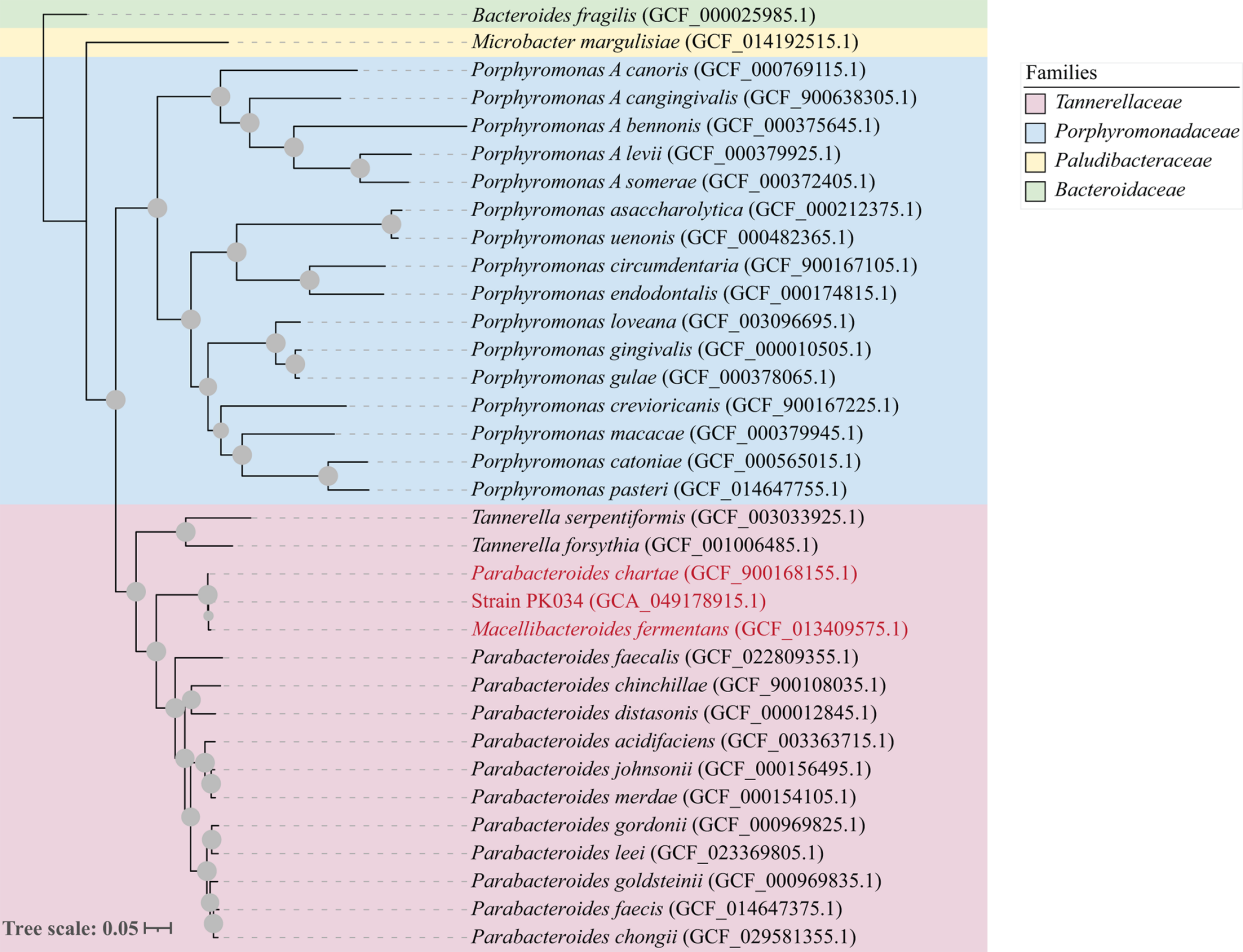
Core-genome phylogenomic tree using a concatenated alignment of 120 single-copy gene sequences showing the relationship among PK034, *M. fermentans* and *P. chartae* among other species of the genus *Parabacteroides*. Bar, 0.05 substitutions per site.

OGRIs for species delineation revealed ANI values of ≥97.5% (Table 2) and dDDH values ≥79.4% (Table 3). These values are higher than proposed thresholds for species-level delineation (95.0–96.0 and 70%, respectively) [32,40, 41]. Similarly, whole-genome phylogenomics (Fig. 2) confirmed the close relationship between PK034 and the type strains of *M. fermentans* and *P. chartae* observed in the single gene 16S rRNA and HSP60 trees (Fig. 1a, b). On the other hand, OGRIs for genus delineation revealed AAI and POCP values ≤68.1% and ≤69.4% between all three isolates on one hand and all other members of the genus *Parabacteroides* on the other (Table 4). Such delineation is further confirmed by the distinct position of all three isolates when compared to all other members of the genus *Parabacteroides* in phylogenomic analysis (Fig. 2).

**Table 3.** Species-level delineation metrics based on whole-genome sequences for PK034, *M. fermentans* and *P. chartae*

ANI (%)				
**Taxa**	**WGS accession**	**PK034**	***M. fermentans***	***P. chartae***
**PK034**	PRJNA1240326	100.0	97.9	97.9
***M. fermentans***	GCF_013409575.1	97.5	100.0	97.9
***P. chartae***	GCF_900168155.1	97.9	97.5	100.0
**dDDH (%**)				
**Taxa**	**WGS accession**	**PK034**	***M. fermentans***	***P. chartae***
**PK034**	PRJNA1240326	100.0	79.4	81.0
***M. fermentans***	GCF_013409575.1	79.4	100.0	79.5
***P. chartae***	GCF_900168155.1	81.0	79.5	100.0

**Table 4.** Genus-level delineation metrics based on whole-genome sequences for PK034, *M. fermentans*, *P. chartae* and other *Parabacteroides* spp

AAI (%)				
**Taxa**	**WGS accession**	**PK034**	***M. fermentans***	***P. chartae***
**PK034**	PRJNA1240326	100.0	96.9	97.6
***M. fermentans***	GCF_013409575.1	96.9	100.0	97.1
***P. chartae***	GCF_900168155.1	97.6	97.1	100.0
*P. acidifaciens*	GCF_003363715.1	66.9	66.9	66.9
*P. chinchillae*	GCF_900108035.1	67.5	67.7	68.1
*P. chongii*	GCF_029581355.1	66.0	66.0	66.0
*P. distasonis*	GCF_000012845.1	66.9	67.0	67.5
*P. faecalis*	GCF_022809355.1	64.3	64.4	64.7
*P. faecis*	GCF_014647375.1	66.3	66.6	66.4
*P. goldsteinii*	GCF_000969835.1	66.4	67.0	66.7
*P. gordonii*	GCF_000969825.1	66.9	67.0	67.1
*P. johnsonii*	GCF_000156495.1	66.7	66.8	67.4
*P. leei*	GCF_023369805.1	66.0	67.0	66.0
*P. merdae*	GCF_000154105.1	67.1	67.3	67.7
**POCP (%**)				
**Taxa**	**WGS accession**	**PK034**	***M. fermentans***	***P. chartae***
**PK034**	PRJNA1240326	100.0	86.9	85.7
***M. fermentans***	GCF_013409575.1	86.7	100.0	86.2
***P. chartae***	GCF_900168155.1	85.5	86.1	100.0
*P. acidifaciens*	GCF_003363715.1	68.0	66.8	67.8
*P. chinchillae*	GCF_900108035.1	69.4	68.4	69.0
*P. chongii*	GCF_029581355.1	66.8	65.6	66.0
*P. distasonis*	GCF_000012845.1	68.3	67.4	68.2
*P. faecalis*	GCF_022809355.1	63.9	64.1	65.7
*P. faecis*	GCF_014647375.1	69.3	67.7	68.8
*P. goldsteinii*	GCF_000969835.1	67.4	65.9	66.8
*P. gordonii*	GCF_000969825.1	66.2	64.6	64.9
*P. johnsonii*	GCF_000156495.1	64.9	63.3	63.1
*P. leei*	GCF_023369805.1	66.7	65.6	66.0
*P. merdae*	GCF_000154105.1	68.1	67.2	66.4

## Comparative phenotypic and physiological analysis

For additional confirmation of the relatedness of strain PK034 and the type strains of *M. fermentans* and *P. chartae*, morphological and physiological characterization of strain PK034 was conducted. The Gram-stain reaction for strain PK034 was determined using the Gram Stain Kit (Becton Dickinson, Franklin Lakes, NJ, USA) following the manufacturer’s instructions. Phase contrast microscopy (BX51, Olympus, Center Valley, PA, USA) was used to inspect cells for shape, size, motility and endospore production. Aerotolerance was determined by placing cultures on BBE under aerobic conditions for 1 week at 37 °C. Temperature ranges for growth were determined in Tryptic soy broth (TSB) at 4 °C and between 20 and 50 °C in increments of 10 °C; salt tolerance was determined in TSB at 0.5% (w/v) and between 1.0% (w/v) and 10.0% (w/v), in increments of 1.0% (w/v); pH ranges for growth were determined in TSB between 3.0 and 10.0, in increments of 1.0. Optimal growth conditions were determined from OD_600_ using a spectrophotometer (Spectronic 20D+, Milton Roy, Houston, TX, USA). An increase in OD_600_ >0.1 after 5 days of incubation was considered growth. Additional characteristics, e.g. substrate utilization data, were obtained from *M. fermentans* and *P. chartae* taxon descriptions manuscripts [23,42].

Strain PK034 is a non-motile, Gram-stain-negative, non-spore-forming, rod-shaped obligate anaerobe, similar to all members of the order *Bacteroidales*. Strain PK034 and the type strains of *M. fermentans* and *P. chartae* all exhibited a comparable temperature (10–40 °C, optimum ~37 °C), pH (5.0–8.5, optimum ~7.0) and salinity (0.0–2.0%, optimum ~0.0 %) (Table 5). As well, a broad concordance in *M. fermentans* and *P. chartae* substrate utilization patterns (Table S1, available in the online Supplementary Material). The close morphological and phenotypic similarity mirrors the high phylogenetic and phylogenomic similarity observed among all three isolates.

**Table 5.** Comparison of morphological characteristics and physiological optima for PK034, *M. fermentans* and *P. chartae*

Characteristic	PKO34	*M. fermentans*	*P. chartae*
**Cell shape**	Rod	Rod	Rod
**Gram-stain reaction**	–	–*	–
**Endospore formation**	–	–	–
**Motility**	–	–	–
**Oxygen requirement**	Obligately anaerobic	Obligately anaerobic	Obligately anaerobic
**Temperature range (°C) (optimum, °C**)	20–40 (37)	20–45 (35–40)	10–40 (35–37)
**pH range (optimum**)	5.0–8.0 (7.0)	5.0–8.5 (6.5–7.5)	5.5–8.5 (7.0–7.5)
**NaCl tolerance (optimum**)	0.5–2.0 (0.5)	0.0–2.0 (nr)†	0.0–2.0 (0.0)

*In the original species description manuscript [23], the result of the Gram-stain reaction for *M. fermentans* was positive. However, the authors confirmed that *M. fermentans* has a cell wall structure that corresponds to Gram-stain-negative bacteria by conducting thin-section electron micrographs.

†nr: Not reported.

## Global ecological distribution patterns of the genera *Macellibacteroides* and *Parabacteroides*

Finally, the ecological distribution patterns of members of the genera *Macellibacteroides* and *Parabacteroides* were assessed using Sandpiper [43] (https://sandpiper.qut.edu.au/), an interface utilizing the SingleM tool for accurate mapping of metagenomic reads to genomes. At the time of analysis, 707,499 different metagenomic datasets (from 32,370 projects) were analysed and available through Sandpiper with taxonomy annotations matching GTDB version R226. Results were downloaded and used to assess the global patterns of distribution (occurrence and relative abundance) and habitat preferences for the genera *Macellibacteroides* and *Parabacteroides*. For assessments of the distribution of the genus *Macellibacteroides*, we used *M. fermentans*, the only species in the genus. For assessment of the genus *Parabacteroides*, we used all 12 species (excluding *P. chartae*).

Our results identified members of the genus *Macellibacteroides* in 9,093 datasets (1.29% of total datasets analysed in Sandpiper). The highest number of datasets was derived from engineered environments (72.2% of total), followed by host-associated (7.06%) and freshwater (5.72%) environments. The highest number of engineered datasets with *Macellibacteroides* hits was derived from wastewater treatment plants (81.3%), indicating a strong preference of members of *Macellibacteroides* to wastewater. The percentage abundance of *Macellibacteroides* varied greatly between datasets, with the highest abundance of 52.51% derived from a lab-scale methane-producing bioreactor metagenome (PRJNA817690). On the other hand, the genus *Parabacteroides* was identified in 257,388 total datasets. The highest number was derived from host-associated environments (97.3%). The percentage abundance varied greatly between datasets, with the highest abundance of 99.93% derived from the human gut metagenome (PRJEB32631).

## Conclusions

Collectively, our phylogenetic (Fig. 1, Table 1), phylogenomic (Fig. 2, Tables2^4^) and physiological characterization (Table 5) results indicate that PK034, *M. fermentans* and *P. chartae* constitute the same species. We propose consolidating them into a single species, *M. fermentans*. The high level of divergence (Figs1  2, Table 4) between *M. fermentans* and *P. chartae*, when compared to all other members of the genus *Parabacteroides*, justifies their distinction from all other taxa in the genus *Parabacteroides*. Suggesting a new genus, or a new combination to accommodate all three isolates, is unwarranted, since the currently validly named genus *Macellibacteroides* [23] appropriately accommodates all three strains. It is also worth mentioning that, in addition to the strong evidence demonstrating that *P. chartae* is erroneously assigned to the genus *Parabacteroides*, priority publication exists for *M. fermentans* over *P. chartae*. According to Rule 24b of the International Code of Nomenclature of Prokaryotes [44], in the event of heterotypic synonyms such as the ones considered in this study, priority shall be given to the name or epithet that is earliest validly published. *M. fermentans* was published on 1 October 2012 by Jabari *et al.* [23] and hence has priority over *P. chartae* Tan *et al.* 2012, published on 1 November 2012 [42]. Therefore, *P. chartae* Tan *et al.* 2012 should be considered a later heterotypic synonym of *M. fermentans* Jabari *et al.* 2012.

## Emended description of *Macellibacteroides fermentans*

The description is as before [23] with the following modifications.

The type strain is LIND7H^T^ (=CCUG 60892^T^=DSM 23697^T^=JCM 16313^T^), and strains NS31-3 (=JCM 17797=DSM 24967) and PK034 are additional strains. The additional strains of *M. fermentans* grow between 10 and 40 °C, pH 5.5–8.5. Strains grow in NaCl concentrations up to 2.0% (w/v). The utilization of glycerol, rhamnose and salicin between strains is variable.

The emended DNA G+C content (mol%) is 41.4–41.9. The additional genome sequence is deposited in GenBank under BioProject number PRJNA1240326. The genome size ranges from 3.9 to 4.3 Mb.

## Supplementary material

10.1099/ijsem.0.006927 Uncited Table S1.

## Abbreviations

ANI: average nucleotide identity
BBE: *Bacteroides* Bile Esculin
dDDH: digital DNA–DNA hybridization
gDNA: genomic DNA
GTDB: Genome Taxonomy Database
OGRIs: overall genomic relatedness indices
POCP: percentage of conserved protein
TSB: Tryptic soy broth
