# Supplementary material for: Genome-based reclassification of Parabacteroides chartae as a later heterotypic synonym of Macellibacteroides fermentans
Source: Int J Syst Evol Microbiol. 2025 Sep 25;75(9):006927. doi: 10.1099/ijsem.0.006927 (PMC13154670; doi:10.1099/ijsem.0.006927)
Supplement: Uncited Table S1. [file ijsem-75-06927-s001.pdf]

Supplementary material

Genome-based reclassification of *Parabacteroides chartae* as a later heterotypic synonym of *Macellibacteroides fermentans*

Authors: Samuel L. Miller, Noha H. Youssef, Mostafa S. Elshahed

Supplementary Table 1: Comparison of Substrate utilization patterns between *Macelibacteroidesoides fermentans* and *Parabacteroides chartae*. Data are from references (23, 40).

Substrate	*M. fermentans*	*P. chartae*
**Arabinose**	+	+
**Cellobiose**	+	+
**Gelatin**	+	+
**Glucose**	+	+
**Glycerol**	+	-
**Lactose**	+	+
**Maltose**	+	+
**Mannitol**	+	+
**Mannose**	+	+
**Melezitose**	+	+
**Raffinose**	+	+
**Rhamnose**	+	-
**Salicin**	-	+
**Sorbitol**	+	+
**Sucrose**	+	+
**Trehalose**	+	+
**Xylose**	+	+

*Denotes that *M. fermentans* has a cell wall structure that corresponds to Gram-stain-negative bacteria. +, Positive; −, Negative.
