# Supplementary material for: Genome-based reclassification of Parabacteroides chartae as a later heterotypic synonym of Macellibacteroides fermentans
Source: Int J Syst Evol Microbiol. Author manuscript; Available in PMC 2026 May 8. (PMC13154670; doi:10.1099/ijsem.0.006927)
Supplement: Supplementary file 1 [file NIHMS2164201-supplement-Supplemental.docx]

**Supplemental Figure 1:** Scanning electron micrograph of P3C3^T^. 2 µm

**Supplemental Figure 2:** Scanning electron micrograph of MAC6^T^1 µm
